# Association between quantitative varicella-zoster virus antibody levels and zoster reactivation in HIV-infected persons

**DOI:** 10.1186/s12981-018-0212-0

**Published:** 2018-12-11

**Authors:** Heather S. Pomerantz, Xiaohe Xu, James White, T. S. Sunil, Robert G. Deiss, Anuradha Ganesan, Brian K. Agan, Jason F. Okulicz

**Affiliations:** 1 Infectious Disease Service, San Antonio Military Medical Center, San Antonio, USA; 2 School of Public Administration, Sichuan University, Chengdu, China; 3 Department of Sociology, University of Texas San Antonio, San Antonio, TX USA; 4 TX Institute for Health Disparities Research, University of Texas at San Antonio, San Antonio, TX USA; 5 Division of Infectious Diseases, Naval Medical Center San Diego, San Diego, CA USA; 6 Infectious Disease Clinical Research Program, Preventive Medicine and Biostatistics Department, Uniformed Services University, Bethesda, MD USA; 7 Henry M. Jackson Foundation for the Advancement of Military Medicine, Bethesda, MD USA; 8 Walter Reed National Military Medical Center, Bethesda, MD USA

**Keywords:** HIV, Varicella zoster virus, Military

## Abstract

**Background:**

Varicella-zoster virus (VZV) reactivation is common but difficult to predict in HIV-infected persons.

**Objective:**

Since qualitative VZV antibodies can determine past VZV disease or vaccination, we evaluated whether quantitative VZV antibody levels over time can predict future zoster.

**Study design:**

US Military HIV Natural History (NHS) participants with a zoster diagnosis at least 5 years after HIV diagnosis (n = 100) were included. Zoster-negative controls (n = 200) were matched by age, race, gender, and CD4 count at HIV diagnosis. Repository plasma specimens collected at baseline and prior to zoster diagnosis were evaluated using a quantitative anti-VZV ELISA assay. Differences in quantitative VZV levels were analyzed by Wilcoxon Mann–Whitney and Fisher’s exact tests.

**Results:**

Median CD4 count at HIV diagnosis was similar for cases and controls (535 [IQR 384–666] vs. 523 [IQR 377–690] cells/μL; p = 0.940), but lower for cases at zoster diagnosis (436 [IQR 277–631] vs. 527 [IQR 367–744] cells/μL; p = 0.007). Antiretroviral therapy (ART) use prior to zoster diagnosis was lower for cases (52.0%) compared to controls (64.5%; p = 0.025). Cases had similar mean VZV antibody levels prior to zoster diagnosis compared to controls [2.25 ± 0.85 vs. 2.44 ± 0.96 index value/optical density (OD) ratio; p = 0.151] with no difference in the change in antibody levels over time (0.08 ± 0.71 vs. 0.01 ± 0.94 index value/OD per year; p = 0.276).

**Conclusion:**

Quantitative VZV antibody levels are stable in HIV-infected persons and do not predict zoster reactivation. Low CD4 count and lack of ART use appear to be better predictors of future zoster diagnosis.

## Background

Infection with varicella zoster virus (VZV) can cause relapse of disease in those with impaired cell mediated immunity (CMI) known as herpes zoster (HZ) and is seen more frequently in those with HIV infection compared to HIV-uninfected individuals [1]. Overall incidence has decreased with increased use of antiretroviral therapy (ART), but rates still remain high in the HIV-infected population compared to the general population (628–650 per 100,000 person years [1, 2] vs. 150–300 per 100,000-person-years [3, 4] respectively). Complications of HZ include bacterial superinfection, post-herpetic neuralgia (PHN), and ocular complications, among others [4]. PHN is a longterm neuropathic pain syndrome that is difficult to treat and it is estimated to affect 10–20% of those with HZ with risk increasing with age [5].

Cell mediated immunity appears to play a role in preventing HZ, as waning CD4 count is associated with development of HZ [1, 4]. Conversely, effective ART results in immune reconstitution and CD4 gains which reduce the risk of VZV reactivation [6, 7]. Even though the association between reduced CMI and VZV reactivation has been well established, it remains difficult to predict disease, as it can occur at any CD4 count, and the risk of HZ remains high in the HIV-infected population even in the ART era [1]. B cell dysfunction has also been observed in those with HIV [8, 9] and can lead to altered antibody response [10]. Clinicians currently use VZV antibody status to determine prior exposure to VZV and help guide vaccine decisions while Varicella vaccine clinical trials have used antibody levels to determine efficacy [11]. It is unclear if these antibody levels wane over time, and what role HIV infection plays in longitudinal quantitative VZV antibody levels.

## Objectives

We retrospectively evaluated VZV antibody titers in HIV-infected persons with and without HZ in order to determine if VZV antibody levels can be used to predict HZ.

## Study design

The US Military HIV Natural History Study (NHS) is a prospective observational study of HIV-infected military members and beneficiaries. Enrolled participants are ≥ 18 years of age with documented HIV infection and complete informed consent in this IRB approved study. NHS participants have clinical visits approximately every 6–12 months at select military treatment facilities.

The NHS database was queried for participants with (cases) or without (controls) a HZ diagnosis after HIV diagnosis. In order to assess the change in serum VZV antibody titers over time, only participants with a HZ diagnosis greater than or equal to 5 years after HIV diagnosis were included. Participants were required to have a serum sample 30–180 days prior to HZ diagnosis in addition to a sample at least 3 years prior to documented HZ infection. A total of 100 cases and 200 controls meeting inclusion criteria and serum sample availability were selected for analysis. Control participants who did not develop HZ were matched by age, race, gender, and CD4 count at time of HIV diagnosis. Participants with negative VZV antibody or receipt of varicella or zoster vaccines were excluded.

Two repository plasma specimens were evaluated using a quantitative anti-VZV ELISA assay (Zeus Scientific, Somerville, NJ). VZV antibody levels are represented for each sample by a calibration cut-off value which is expressed as index value/OD ratio. Specimen time-points for controls were matched to corresponding cases. Differences in quantitative VZV levels were analyzed by the Wilcoxon Mann–Whitney test and Fisher’s Exact test, controlling for time intervals between specimens.

## Results

Participants were predominantly male (94%) with a median age of 29 [IQR 24–34] years at HIV diagnosis (Table 1). The median CD4 count at HIV diagnosis was similar for cases and controls (535 [IQR 384–666] vs. 523 [IQR 377–690] cells/μL; p = 0.940). Cases had a significantly lower CD4 count at the second serum date compared to controls (436 [IQR 277–631] vs. 527 [IQR 367–744] cells/μL; p = 0.007). ART use at HZ diagnosis was also lower at the second serum date for cases compared to controls (52% vs. 64.5%; p = 0.025). At HZ diagnosis, 60% of cases were on ART. The median CD4 count and viral load (VL) at time of VZV diagnosis were 443 cells/μL and 3.51 log_10_ copies/mL, respectively.

**Table 1:** Baseline characteristics of military personnel with HIV

Characteristics	All N = 300	Cases N = 100	Control N = 200	p-value
Median age at HIV diagnosis (years)	28.35	28.35	28.25	0.978
Median time HIV negative to positive (years)	0.68	0.66	0.69	0.828
Median time HIV diagnosis to 1st ART (years)	5.41	5.78	4.82	0.078
Gender				0.611
Male	282 (94%)	94 (94%)	188 (94%)	
Female	18 (6%)	6 (6%)	12 (6%)	
Race/ethnicity				0.024
Caucasian	134 (44.7%)	47 (47%)	87 (43.5%)	
African–American	123 (41%)	36 (36%)	87 (43.5%)	
Hispanic/Puerto Rican/Mexican	28 (9.3%)	13 (13%)	15 (7.5%)	
Asian/Pacific Islander	5 (1.7%)	0	5 (2.5%)	
Native American/Alaskan Native	3 (3%)	3 (3%)	0	
Other	7 (2.3%)	1 (1%)	6 (3%)	
Median time HIV diagnosis to last study visit (years)	13.18	14.28	13.0384	0.201
Median time from HIV diagnosis to 1st serum date (years)	2.18	1.38	2.53	0.009
Median time from HIV diagnosis to 2nd serum date (years)	6.89	6.98	6.81	0.975
Median CD4 at 1st serum date (cells/μL)	530	534.5	523	0.940
Median VL at 1st serum date (log10 copies/mL)	3.66	3.72	3.65	0.471
Median CD4 at 2nd serum date (cells/μL)	501	436	527	0.007
Median VL at 2nd serum date (log10 copies/mL)	2.80	3.52	2.60	0.007
On ART at 1st serum date				0.075
No	201 (67%)	73 (73%)	128 (64%)	
Yes	99 (33%)	27 (27%)	72 (36%)	
On ART at 2nd serum date				0.025
No	119 (39.7%)	48 (48%)	71 (35.5%)	
Yes	181 (60.3%)	52 (52%)	129 (64.5%)	

Cases had similar mean VZV antibody levels prior to HZ diagnosis compared to controls (2.25 ± 0.85 vs. 2.44 ± 0.96 index value/OD; p = 0.151) and there was no difference in the change in VZV antibody levels over time (0.08 ± 0.71 vs. 0.01 ± 0.94 index value/OD per year; p = 0.276). The range of values for cases was similar to controls [IQR 1.7–2.9 index value/OD vs. 1.7–3 index value/OD]. However, the mean antibody level was higher for those on ART (2.48 index value/OD) compared to those who were not on ART (2.26 index value/OD) with SD of 0.89 and p < 0.05 (Table 2). When evaluating the change in antibody level over time, those with HIV diagnosis for greater than 7 years had a small but significant increase in antibody level over time by 0.03 index value/OD per year compared to those with a diagnosis of HIV for less than 7 years (decrease of 0.04 index value/OD per year) (Table 3).

**Table 2:** VZV level prior to zoster diagnosis subgroup analysis

	Mean index value/OD (n)	SD	T-test
On ART at second serum			
No	2.26 (119)	0.89	− 2.08*
Yes	2.48 (180)	0.93	− 2.08*
Duration of HIV infection at second serum (years)			
< 7	2.38 (153)	0.95	− 0.30
7+	2.41 (146)	0.89	− 0.30
Viral load at second serum (log10 copies/mL)			
< 400	2.41 (127)	0.87	0.20
400+	2.38 (170)	0.95	0.20
CD4 at second serum (cells/μL)			
< 500	2.42 (149)	0.97	0.56
500+	2.36 (149)	0.87	0.56
CD4 at HIV diagnosis (cells/μL)			
< 500	2.45 (111)	0.88	0.90
500+	2.34 (157)	0.97	0.90
Age at HIV diagnosis (years)			
< 30	2.40 (174)	0.98	0.09
30+	2.39 (125)	0.82	0.09
Age at VZV infection (years)			
< 40	2.27 (59)	0.97	0.32
40+	2.22 (40)	0.65	0.32

* indicates p < 0.05

**Table 3:** Average VZV level change per year subgroup analysis

	Mean index value/OD (n)	SD	T-test
On ART at second serum			
No	0.03 (119)	0.21	1.58
Yes	− 0.01 (180)	0.25	1.58
Duration of HIV infection at second serum (years)			
< 7	0.04 (153)	0.27	2.45*
7+	− 0.03 (146)	0.19	2.45*
Viral load at second serum (log10 copies/mL)			
< 400	0.01 (127)	0.21	− 0.01
400+	0.01 (170)	0.25	− 0.01
CD4 at second serum (cells/μL)			
< 500	− 0.01 (149)	0.27	− 1.02
500+	0.02 (149)	0.19	− 1.02
CD4 at HIV diagnosis (cells/μL)			
< 500	0.00 (111)	0.25	− 0.09
500+	0.01 (157)	0.22	− 0.09
Age at HIV diagnosis (years)			
< 30	0.01 (174)	0.25	− 0.02
30+	0.01 (125)	0.21	− 0.02
Age at VZV infection (years)			
< 40	0.01 (59)	0.17	0.12
40+	0.01 (40)	0.14	0.12

* indicates p < 0.05

## Discussion

Although VZV antibody can identify exposure to Varicella infection or past immunization, we found that neither the magnitude of VZV antibody level nor the change in concentration over time predicted HZ reactivation in HIV-infected persons. CMI most likely plays a larger role in immune protection from HZ and may be a better predictor of HZ than VZV antibodies, however the latter have been shown to be important as described below. Markers of intact CMI are associated with reduced incidence of HZ and reduced PHN [12, 13]. The zoster vaccine increases the number of VZV specific CD4+ T cells [14] and older adults have an increase in IFNγ after a second dose of vaccine [15]. However, the only marker of CMI assessed clinically is CD4 count which is associated with development of HZ but not always predictive. For example, a higher prevalence of HZ has been observed in those with VL suppression and CD4 > 750 cells/μL compared to HIV-uninfected persons [1, 7, 16].

In addition to a T-cell mediated immune response, humoral response also appears to be important as VZV antibodies have been used to demonstrate vaccine effectiveness [11, 17–19]. A study analyzed gpELISA VZV antibody prior to immunization and at week 6 after immunization and found that 100% rise (onefold) in levels better correlated with protection against HZ rather than antibody level alone irrespective of baseline level [20]. Additionally, varicella zoster immune globulin (VZIG) is given to allow passive immunity and is currently recommended in those with high risk exposure [21]. Currently there is a licensed vaccine to prevent the occurrence of HZ and reduce the complications such as PHN [17, 21]. However, this is only FDA approved for those over age 50 and is contraindicated in HIV infected individuals with a CD4 count less than 200 cells/μL [21, 22].

In the population with HIV infection, the risk for HZ exists even in those less than the age of 40 [6] and it would be helpful to have a marker that can predict who will develop HZ as they may benefit from vaccination even if not in the current recommended age range. Although our study showed no association between VZV antibody levels and clinical HZ in an HIV-infected population, we validated findings from prior studies demonstrating that that lower CD4 count and lack of ART use were risk factors for the development of HZ. Other risk factors described in this population include high VL and prior history of HZ [6, 7]. In order to reduce the morbidity associated with HZ in HIV-infected persons, additional clinical markers of risk are needed as well as VZV vaccine safety and efficacy studies in younger patients with preserved CD4 counts.
